# A Comprehensive Pan-Cancer Analysis of RBM8A Based on Data Mining

**DOI:** 10.1155/2021/9983354

**Published:** 2021-07-06

**Authors:** Nan Mei, Heyan Chen, Ni Zhao, Ye Yi, Chunli Li

**Affiliations:** ^1^Department of Medical Oncology, The First Affiliated Hospital of Xi'an Jiaotong University, Xi'an, Shaanxi Province, China; ^2^Department of Breast Surgery, The First Affiliated Hospital of Xi'an Jiaotong University, Xi'an, Shaanxi Province, China

## Abstract

**Background:**

As a member of the exon junction complex (EJC), RNA-binding motif protein 8A (RBM8A) plays a crucial role in the maintenance of mRNA and multiple activities of an organism. Immunotherapy has been proven to be a staple type of cancer treatment. However, the role of RBM8A and immunity across cancer types is unclear.

**Objective:**

This study aims to visualize the expression, prognosis, mutations, and coexpressed gene results of RBM8A across cancer types and to explore the link between RBM8A expression and immunity.

**Methods:**

In this study, data were collected from multiple online databases. We analyzed the data using the HPA, UALCAN Database, COSMIC, cBioPortal, Cancer Regulome tools, Kaplan–Meier Plotter, and TIMER website.

**Results:**

For the expression of RBM8A in normal tissues, higher expression of RBM8A was observed in immune-related cells than in nonimmune organs. The expression level of RBM8A was related to tumor type. Missense mutations in RBM8A were found in most tumors and affected the prognosis of carcinomas with coexpressed genes. RBM8A was strongly associated with immune-infiltrating cells and immune checkpoint inhibitors, especially in LIHC.

**Conclusions:**

RBM8A is a gene worth exploring and may be a unique immune target in the future.

## 1. Introduction

Cancer is an incurable disease and therefore has become a major global cause of morbidity and mortality. The reported figures for the 2018 Global Cancer Estimate have indicated 18.1 million new cancer cases and 9.6 million deaths from cancer in 2018 [1]. Although the progress of medical technology, specifically the combined application of multiple treatment methods, has prolonged the survival rate of cancer patients and improved their quality of life, in order to further benefit cancer patients, it is necessary to constantly develop new drugs and explore more effective treatment measures. In recent years, the emergence and application of immunotherapy have revolutionized the treatment of many cancers. In oncology immunotherapy, the discovery and clinical application of checkpoint inhibitors have greatly accelerated the process of immunotherapy. The cytotoxic T lymphocyte antigen 4 (CTLA4) or programmed cell death 1 (PD-1)/PD-1 ligand 1 (PD-L1) axis is approved for use in a variety of cancer types [2]. After clinical application, it has been found that immune checkpoint inhibitors are not effective for all cancer populations, and some may even lead to serious immune-related adverse events in patients [3, 4]. Therefore, immunotherapy, such as targeted therapy, requires biomarkers to predict efficacy in advance. Studies [5] have shown that immune-infiltrating cells are closely related to immunotherapy, and hence, basic knowledge of immune-infiltrating cells is important for the evaluation of the efficacy of immunotherapy.

RNA-binding motif protein 8A (RBM8A), a core factor in the exon junction complex (EJC), plays a number of roles in mRNA metabolism [6, 7]. It is involved in nonsense-mediated mRNA decay (NMD), mRNA translation, and the selective splicing of apoptotic factors. It has been reported [8] that RBM8A deficiency causes irradiated thrombocytopenia-absent radius (TAR) syndrome. In addition to TAR, changes in the level of RBM8A expression can also lead to the occurrence of some types of cancer and affect their prognosis. However, there are very few experimental studies and relevant data on RBM8A and cancer, and no studies on RBM8A and the different types of carcinomas have been found; thus, the mechanism of action is unclear. Therefore, it is important to expand the research on the role of RBM8A in cancer.

In this pan-cancer study, we used a database to comprehensively analyze the expression, mutation, and prognosis of RBM8A and obtained a series of corresponding results. Based on the results, we used the TIMER database to analyze the correlation between the expression of RBM8A and immune-infiltrating cells in multiple types of cancer to further examine the role of RBM8A in immunotherapy. We also used the TIMER database to analyze the correlation between RBM8A expression and immune checkpoints. Our study found that RBM8A may interact with immune-infiltrating cells through signaling pathways in cancer and simultaneously influence immune checkpoints to regulate the occurrence of immune responses.

## 2. Material and Methods

### 2.1. The Human Protein Atlas (HPA)

HPA (http://www.proteinatlas.org) is a database that allows us to study the function of proteins in greater detail. The HPA database uses transcriptomics and proteomics technologies to study protein expression in different human tissues and organs at the RNA and protein levels [9]. In this article, we used this database to determine the level of RBM8A mRNA expression in human tissues.

### 2.2. UALCAN Database

UALCAN (http://ualcan.path.uab.edu) is an online site for analyzing and mining types of cancer associated with The Cancer Genome Atlas (TCGA) database, which helps medical workers analyze the levels of gene expression and obtain survival analysis, correlation analysis, gene promoter methylation data analysis, etc. Therefore, the UALCAN web portal is extremely helpful in accelerating cancer research [10]. In this study, we used the UALCAN database to obtain data from the TCGA database and compared the expression of RBM8A mRNA in tumors and normal tissues.

### 2.3. Catalog of Somatic Mutations in Cancer (COSMIC)

COSMIC (https://cancer.sanger.ac.uk/cosmic/) is a database system designed to provide information about somatic mutations in types of human cancer in a single system and make them easily accessible. COSMIC describes coding gene point mutations, millions of coding mutations, noncoding mutations, genomic rearrangements, fusion genes, copy number abnormalities, and gene expression variants across the human genome [11]. In this study, COSMIC was used to show the mutations of RBM8A in human cancers, and the results are depicted in pie charts.

### 2.4. The cBio Cancer Genomics Portal (cBioPortal)

The cBio Cancer Genomics Portal (http://cbioportal.org) is an open platform for interactive research of all-round cancer genomics datasets in the context of clinical data and biological pathways [12]. We used cBioPortal to analyze RBM8A in TCGA pan-cancer data to identify coexpressed genes.

### 2.5. The Cancer Regulome Tools

The Cancer Regulome (http://explorer.cancerregulome.org/) is a web-based tool that provides data from TCGA. We used cBioPortal to analyze RBM8A in TCGA pan-cancer data to identify coexpressed genes. Spearman's correlation was used to verify the correlation between these two genes. *P* values > −log10.

### 2.6. Kaplan–Meier Plotter

The Kaplan–Meier Plotter (http://kmplot.com/analysis/) is an online tool based on the databases from GEO, EGA, and TCGA. Across cancer types, the samples were divided into high and low groups according to the median value of gene expression, and K–M survival analysis was performed by the Kaplan–Meier Plotter [13]. In this study, we explored the effect of RBM8A expression on OS in different cancer types. Hazard ratios with 95% confidence intervals and log-rank *P* values were calculated simultaneously.

### 2.7. Tumor Immune Estimation Resource (TIMER)

TIMER (http://timer.cistrome.org/) provides comprehensive analysis and visualization functions of tumor-infiltrating immune cells [14, 15]. We analyzed the relationship of RBM8A expression with 6 types of infiltrating immune cells (CD4+ T cells, CD8+ T cells, macrophages, B cells, neutrophils, and dendritic cells) in some types of cancer. We also studied the correlation between RBM8A expression and PD-1, PD-L1, and CTLA4 in specific types of cancer via TIMER2.0.

## 3. Results

### 3.1. RBM8A mRNA in Normal Tissues

**Figure 1:**
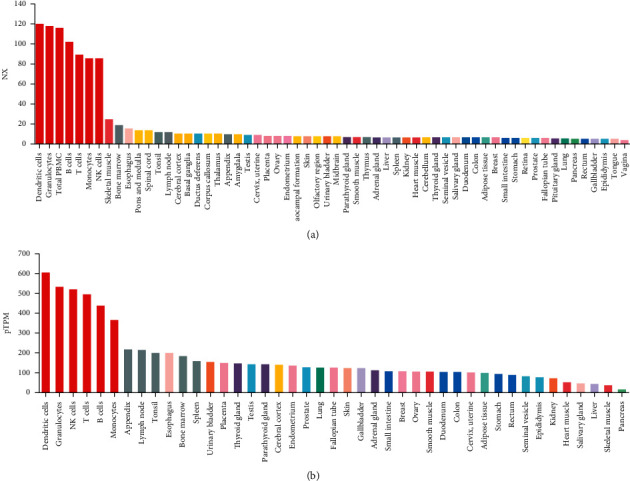
Expression profile for RBM8A mRNA in human different tissues displayed by HPA (Human Protein Atlas). (a) Consensus dataset; (b) HPA dataset.

RBM8A is present in different human tissues. As shown in the results of the Consensus dataset and HPA dataset in Figure 1, compared with other normal tissues, we observed higher expression of RBM8A in B cells, T cells, NK cells, dendritic cells, granulocytes, and monocytes. Therefore, we believe that RBM8A is more highly expressed in immune-related cells than in nonimmune organs.

### 3.2. Expression Level of RBM8A mRNA in Pan-Cancer

**Figure 2:**
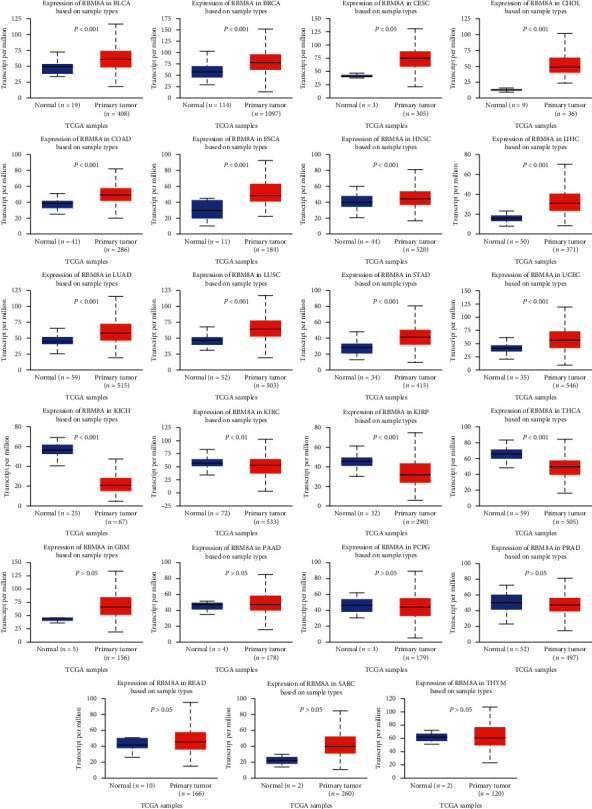
RBM8A mRNA was evaluated in human cancers compared with normal tissues (UALCAN).

To detect the mRNA expression level of RBM8A in diverse carcinoma types, the UALCAN database was used to obtain the relevant data. As shown in Figure 2, compared with that in the corresponding normal groups, RBM8A expression was higher in breast invasive carcinoma (BRCA), bladder urothelial carcinoma (BLCA), cervical squamous cell carcinoma (CESC), cholangiocarcinoma (CHOL), colon adenocarcinoma (COAD), head and neck squamous cell carcinoma (HNSC), esophageal carcinoma (ESCA), liver hepatocellular carcinoma (LIHC), lung adenocarcinoma (LUAD), lung squamous cell carcinoma (LUSC), uterine corpus endometrial carcinoma (UCEC), and stomach adenocarcinoma (STAD). Lower expression of RBM8A was observed in kidney chromophobe (KICH), kidney renal papillary cell carcinoma (KIRP), kidney renal clear cell carcinoma (KIRC), and thyroid carcinoma (THCA). However, no significant differences were found in prostate adenocarcinoma (PRAD), glioblastoma multiforme (GBM), pancreatic adenocarcinoma (PAAD), pheochromocytoma and paraganglioma (PCPG), sarcoma (SARC), rectum adenocarcinoma (READ), or thymoma (THYM).

### 3.3. RBM8A Mutations in Pan-Cancer

**Figure 3:**
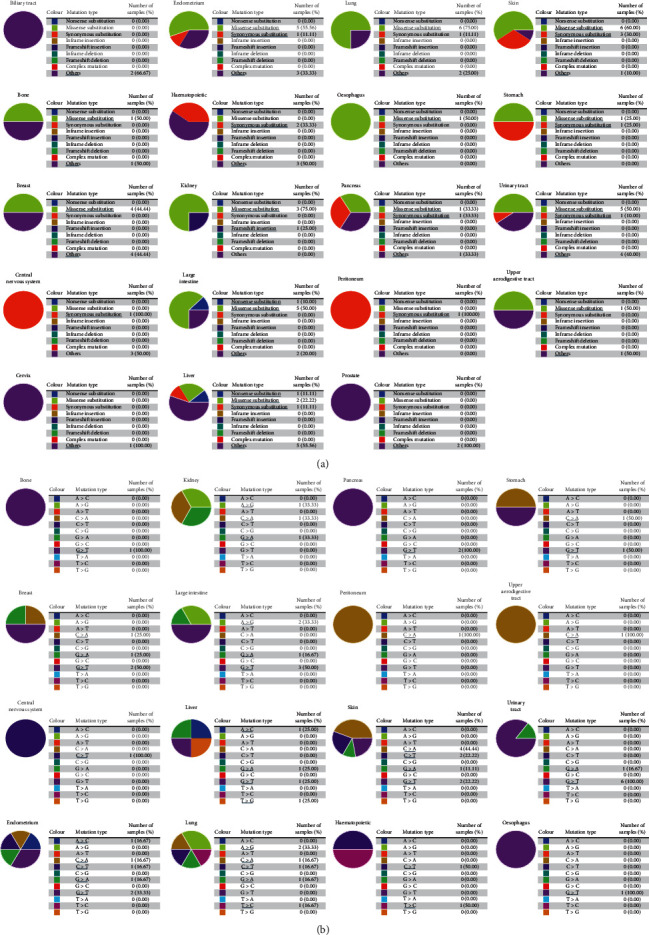
Pie chart showing the percentage of the different mutation types of RBM8A in human cancers (COSMIC). (a) Mutation types of RBM8A. (b) Substitution mutations of RBM8A.

COSMIC provided information on RBM8A mutations in various types of cancer, which included substitution missense mutations, synonymous mutations, and nonsense mutations, and the results are depicted in pie charts. There are no substitution mutations in the adrenal gland or ovary. As shown in Figure 3, missense mutations were found in bone cancer (1%), breast cancer (4%), endometrium (5%), kidney (3%), large intestine (5%), liver cancer (2%), lung cancer (6%), esophagus (1%), pancreas (1%), skin (6%), stomach (1%), upper aerodigestive tract (1%), and urinary tract (5%). Synonymous substitution mutations appeared in the central nervous system (1%), endometrium (1%), hematopoietic system (2%), liver (1%), pancreas (1%), peritoneum (1%), skin (3%), stomach (1%), and urinary tract (1%). Nonsense substitutions were found in the large intestine (1%) and liver (1%). Frameshift insertion was only observed in the kidney (1%). G > T mutations were found in bone (1%), breast (2%), endometrium (2%), large intestine (3%), liver (1%), esophagus (1%), pancreas (2%), skin (2%), stomach (1%), and urinary tract (6%). G > A mutations were found in the breast (1%), endometrium (1%), large intestine (1%), kidney (1%), liver (1%), lung (1%), skin (1%), and urinary tract (1%). C > A mutations were found in the breast (1%), endometrium (1%), kidney (1%), lung (1%), peritoneum (1%), skin (4%), stomach (1%), and upper aerodigestive tract (1%). C > T mutations were found in the central nervous system (1%), endometrium (1%), hematopoietic system (1%), lung (1%), and skin (2%). A > C mutation was found in the endometrium (1%) and liver (1%). A > G mutations were found in the kidney (1%), large intestine (2%), and lung (2%). Other types of mutations occur sporadically in different forms of cancer.

**Figure 4:**
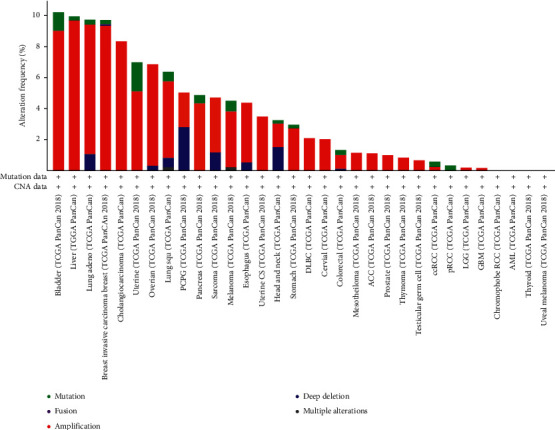
RBM8A mutation level in the TCGA pan-cancer database (cBioPortal).

**Figure 5:**
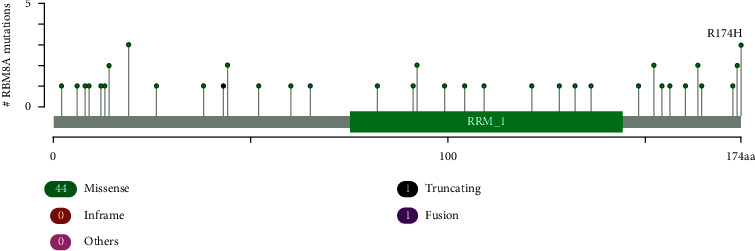
Mutation diagram of RBM8A in different cancer types across protein domains (cBioPortal).

As shown in Figure 4, the TCGA database contained a high level of RBM8A mutation in the following types of cancer: bladder cancer, liver cancer, lung cancer, breast cancer, uterine cancer, pancreas, melanoma, head neck, stomach, colorectal cancer, ccRCC, and pRCC. Through cBioPortal, 44 mutation sites were detected, and they were located between amino acids 0 and 174 (Figure 5).

### 3.4. Genome-Wide Association of RBM8A mRNA in Cancer

**Figure 6:**
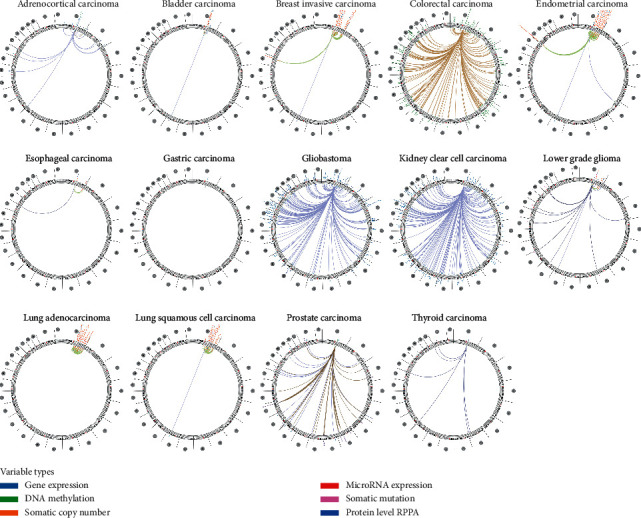
The correlation between RBM8A and other genes from the TCGA database (Regulome program).

Based on the association among genes, somatic copy number, DNA methylation, somatic mutation, and protein level, circus diagrams were drawn to display the interrelation between RBM8A and other genes by using Regulome Explorer. In accordance with the data from TCGA, RBM8A was correlated with other genes that could be detected in adrenocortical carcinoma, breast invasive carcinoma, bladder carcinoma, breast invasive carcinoma, endometrial carcinoma, esophageal carcinoma, gastric carcinoma, glioblastoma, kidney clear cell carcinoma, lung adenocarcinoma, lower-grade glioma, lung squamous cell carcinoma, prostate carcinoma, and thyroid carcinoma (Figure 6). Detailed data are recorded in Supplementary Tables S1–S14.

### 3.5. RBM8A and the Survival Rate of Cancer

**Figure 7:**
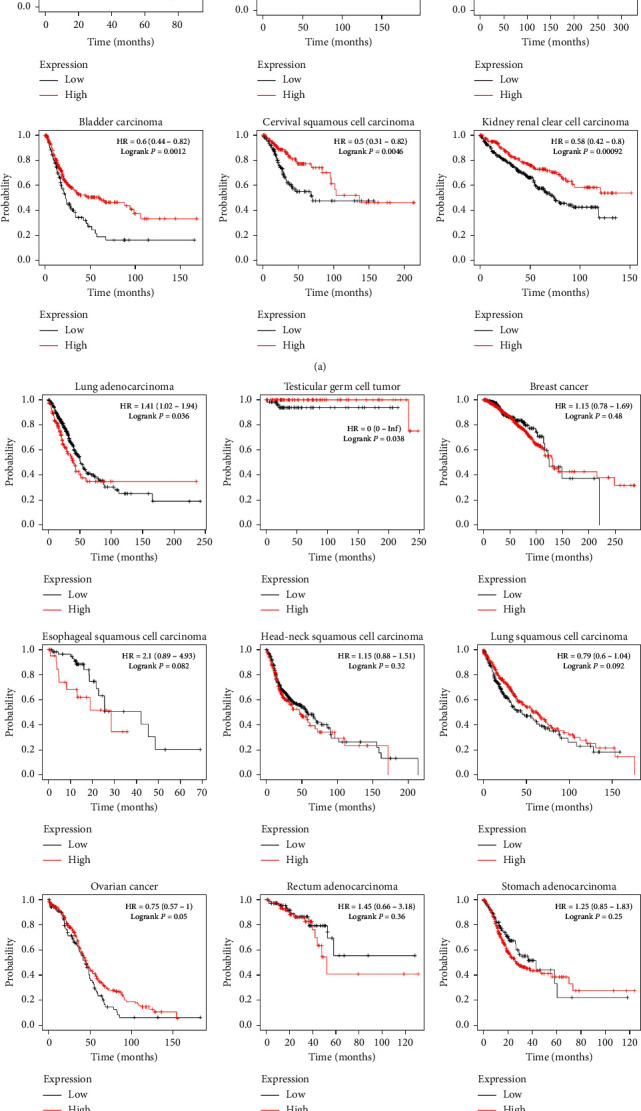
Different expression levels of RBM8A mRNA will result in different overall survival rates in cancers (Kaplan–Meier Plotter).

The relationship between RBM8A gene expression and overall survival (OS) was evaluated by the Kaplan–Meier method combined with the log-rank test. According to the Kaplan–Meier analysis results (Figure 7), higher levels of RBM8A mRNA indicated worse overall survival in esophageal adenocarcinoma (*P* = 0.029), kidney renal papillary cell carcinoma (*P* = 0.044), liver hepatocellular carcinoma (*P* = 0.0085), pancreatic ductal adenocarcinoma (*P* = 0.013), pheochromocytoma and paraganglioma (*P* = 0.042), and sarcoma (*P* = 0.0076). However, the opposite result was observed in bladder carcinoma (*P* = 0.0012), cervical squamous cell carcinoma (*P* = 0.0046), kidney renal clear cell carcinoma (*P* = 0.00092), lung adenocarcinoma (*P* = 0.036), and testicular germ cell tumor (*P* = 0.038). The expression of the RBM8A mRNA level had no significant influence in breast cancer (*P* = 0.48), head neck squamous cell carcinoma (*P* = 0.32), esophageal squamous cell carcinoma (*P* = 0.082), ovarian cancer (*P* = 0.05), stomach adenocarcinoma (*P* = 0.25), rectum adenocarcinoma (*P* = 0.36), thymoma (*P* = 0.28), thyroid carcinoma (*P* = 0.2), or uterine corpus endometrial carcinoma (*P* = 0.088).

### 3.6. Correlations between RBM8A Expression and Immune Cells and Immune Checkpoint Inhibitors in TIMER

**Figure 8:**
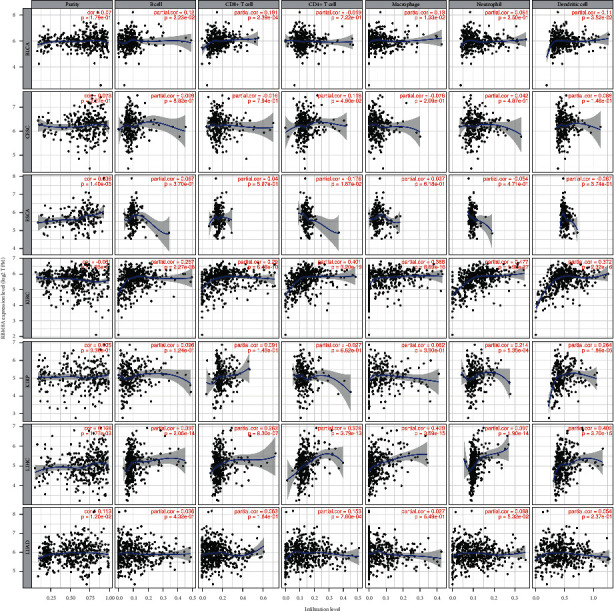
Correlation of RBM8A expression with immune infiltration levels of B cell, CD8+ T cell, CD4+ T cell, macrophage, neutrophil, and dendritic cell in cancers (TIMER) (*p* < 0.05 is considered as significant).

The connection between RBM8A expression and immune infiltration was determined through TIMER. We analyzed RBM8A expression with the abundance of all six types of immune-infiltrating cells, including CD4+ T cells, CD8+ T cells, macrophages, B cells, neutrophils, and dendritic cells. The correlation between gene expression and immune infiltration was estimated by the Pearson correlation test. Based on the above analysis results of RBM8A expression in cancer and survival prognosis, we finally selected 7 types of cancer to analyze the relationship between RBM8A and immune infiltration cells in TIMER, which were ESCA, LIHC, and KIRP to represent cancers with worse survival and BLCA, CESC, KIRC, and LUAD to represent cancers with good survival when RBM8A had a high level of expression. As shown in Figure 8, for LIHC, the level of RBM8A expression had significant positive interactions with the infiltration levels of B cells (*R* = 0.375, *P* = 5.48*e* − 13), CD8+ T cells (*R* = 0.144, *P* = 7.50*e* − 03), CD4+ T cells (*R* = 0.294, *P* = 2.65*e* − 08), macrophages (*R* = 0.345, *P* = 4.74*e* − 11), neutrophils (*R* = 0.243, *P* = 5.21*e* − 06), and dendritic cells (*R* = 0.444, *P* = 4.63*e* − 18). In addition, in KIRP, BLCA, KIRC, and LUAD, the connection between the RBM8A expression level and immune-infiltrating cells was almost the same as that of LIHC. However, for ESCA and CESC, the RBM8A expression level had no relation with the above immune infiltration cells. Next, we used TIMER to explore the correlation between the mutation of RBM8A and immune-infiltrating cells across multiple types of cancer. The results showed that the RBM8A mutant gene had no significance with the 6 infiltrated cells in the pan-cancers (Supplementary Figures S1–S6 and Supplementary Tables S15–S20).

**Figure 9:**
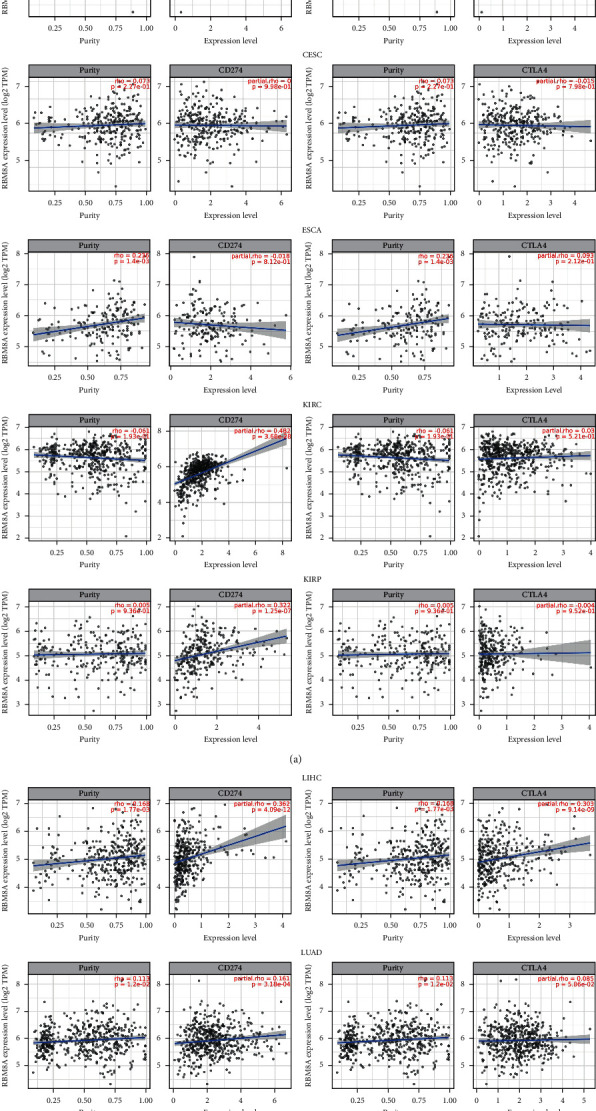
The relationship between RBM8A and immunity (PD-L1 and CTLA4) in different cancers (TIMER).

Based on the results of RBM8A and the immune-infiltrating cells, we chose LIHC, KIRP, BLCA, KIRC, and LUAD to explore the correlation and significance of RBM8A and immune checkpoint inhibitors, such as PD-L1 and CTLA4. According to the images created by TIMER (Figure 9), it is obvious that in LIHC, the RBM8A expression level had significant positive interactions with PD-L1 (*R* = 0.362, *P* = 4.09*e* − 12) and CTLA4 (*R* = 0.303, *P* = 9.14 − 09). In addition, in KIRP and BLCA, the results of the correlation between RBM8A and PD-L1 showed a similar trend as LIHC, whereas no correlation between RBM8A and CTLA4 was found in the two types of cancer. Finally, we found that no significant links were observed in KIRC and LUAD. Therefore, we speculate that RBM8A may be a promising target in cancer immunotherapy, especially in LIHC.

## 4. Discussion

### 4.1. Structure and Biological Action of RBM8A

The processes of gene expression are involved in a range of biological activities, including transcription, RNA splicing, translation, and posttranslational modification of proteins [16]. The exon junction complex (EJC) is mainly connected to newly spliced mRNA, coordinating exonuclear transport, translational control, and nonsense-mediated mRNA decay (NMD), thus acting on the process of gene expression [17]. RNA-binding motif protein 8A (RBM8A), also known as Y14, is a member of the RNA-binding motif protein family. It is widely expressed in cells and mainly localized in the nucleoplasm. RBM8A and MAGOH form a heterodimer, which is an important part of the tetrameric core of the EJC [18]. RBM8A plays an important role in mRNA metabolism. First, RBM8A-MAGOH polymerase activates NMD, eliminates mRNA containing nonsense mutations due to abnormal splicing, and enables normal transcriptional translation of tumor suppressor genes. Second, RBM8A connects EJC-related factors and directly regulates the alternative splicing function of genes, such as specifically regulating the alternative splicing of apoptotic factors; by regulating the alternative splicing of apoptotic factors, RBM8A can enhance or weaken the expression of proapoptotic isomers. Based on the above functional basis, RBM8A is combined with other mRNA regulators to regulate cell activity and the cell cycle by splicing and coupling NMD [19–21]. It has been reported that RBM8A deficiency leads to G2/M phase arrest and cell apoptosis. RBM8A depletion results in cumulative DNA damage and reduced cell viability and proliferation capacity [22, 23].

### 4.2. RBM8A Affects the Occurrence and Development of Malignant Tumors

As Figure 1 shows, compared to normal human tissues, RBM8A is more highly expressed in most immune cell types, including dendritic cells (DCs), B cells, T cells, and human tissues. In view of the existence and characteristics of RBM8A, RBM8A is considered to be a new proto-oncogene. In addition to normal tissues and immune cells, many studies have found that RBM8A is highly expressed in certain cancer tissues. Using public sequencing data, Lin et al.'s team [24] analyzed the expression of RBM8A in HCC and its potential role in the regulatory network. They found that the RBM8A mRNA level and copy number variation (CNV) in HCC were significantly higher than those in normal liver tissue, and the RBM8A gene was often amplified in HCC. Functional network analysis showed that in HCC, the expression of RBM8A was involved in ribosomal signal transduction, RNA transport, mRNA monitoring, and spliceosome signaling and regulated DNA replication, repair, and cell cycle progression through cancer-related kinases. Consistent with the results of Lin et al.'s team, Liang et al.'s team [25, 26] also found that RBM8A was highly expressed in HCC tumor tissues and further proved that RBM8A promoted the migration and invasion of tumor cells in HCC by activating the epithelial-mesenchymal transformation signaling pathway. In addition to liver cancer, studies have also reported that the expression level of RBM8A in gastric cancer and colon adenocarcinoma is higher than that in adjacent tissues, and its expression level is positively correlated with tumor size, depth of invasion, and lymph node metastasis; RBM8A can also be used as an independent prognostic factor that affects the overall survival rate of patients [27, 28]. To study the correlation between the gene expression profile of primary cervical tumor tissue and lymph nodes, Kim et al. [29] screened patients with primary cervical cancer and found that RBM8A was highly expressed in lymph node metastatic lesions compared with those without cervical cancer lymph node metastasis. In addition to the above types of cancer, there are studies reporting that the expression of RBM8A is also upregulated in pleural mesothelioma and non-small-cell lung cancer tumor tissues [30, 31]. In recent years, an increasing number of studies have been conducted on RBM8A and cancers, but no integrative pan-cancer analysis of RBM8A has been found thus far. In this pan-cancer study, we analyzed the expression, mutation, and prognosis of RBM8A using public sequencing data. As shown in Figure 2, we found that RBM8A was highly expressed in BLCA, CHOL, COAD, ESCA, HNSC, and UCEC from the database. Our Kaplan–Meier analysis results show that high expression levels of RBM8A mRNA imply worse overall survival in ESCA, KIRP, LIHC, PAAD, PCPG, and SARC. Our research results are consistent with the current research results, further indicating that changes in the expression of the RBM8A gene are closely related to the occurrence of malignant tumors. RBM8A can be used as a biomarker to predict tumor occurrence and metastasis.

In addition to changes in the expression level of the RBM8A gene, this study also found that RBM8A mutations exist in most cancers. The rates of different mutational processes vary among tumors and cancer types. In the life cycle of human cells, gene mutations are constantly accumulating. Some mutations will not change cell function, and some will cause the original anticancer function of genes to promote oncogenesis [32]. The most important question, then, is whether mutations in RBM8A can affect the development of cancer. The current studies are still superficial, and the relationship between RBM8A mutation and cancer and the mechanism of action have not been clearly described. Therefore, more extensive and in-depth studies are needed to determine the significance of the RBM8A mutation in oncogenesis.

### 4.3. RBM8A Regulates the Role of Immune Cells in Tumors through Signaling Pathways

The treatment of tumors has always been a major challenge in the medical field. Surgery, cytotoxic chemotherapy, molecular targeted therapy, and antiangiogenesis therapy have been constantly changing and improving, but there are still problems with tolerance and efficacy. In addition to the above treatment strategies, various forms of cancer immunotherapy, including soluble tumor virus therapy, cancer vaccination, cytokine therapy, adoptive transfer sex cells, and immune checkpoint inhibitors, have risen to prominence in the field of antitumor research and application [33, 34]. In particular, the use of cytotoxic T lymphocyte antigen 4 (CTLA4), programmed cell death 1 (PD-1), and programmed cell death 1 ligand 1 (PD-L1) inhibitors in the clinic has become a landmark breakthrough in tumor immunotherapy [35]. Because of acquired resistance and tumor immune escape, immunotherapy continues to face huge difficulties in achieving a high and sustained response rate in cancer patients. Some studies [36] believe that the understanding of tumor infiltrating lymphocytes (TILs) and the tumor immune microenvironment (TIME) may improve existing immunotherapies, thereby enabling cancer patients to obtain better clinical treatment effects. Immune-infiltrating cells are an important component of the tumor microenvironment, mainly including B cells, CD4+ T cells, CD8+ T cells, neutrophils, macrophages, and dendritic cells, which have been proven to influence the immunotherapy response and promote tumor progression [37–39]. According to the above pan-cancer visualization analysis results of RBM8A, the expression of RBM8A is significant in ESCA, LIHC, KIRP, BLCA, CESC, KIRC, and LUAD and is related to the prognosis of these carcinomas. To further study the correlation between RBM8A and immune-infiltrating cells, we selected the above types of cancer and obtained the corresponding results. Figure 8 clearly shows that RBM8A is positively correlated with immune-infiltrating cells in LIHC, KIRP, BLCA, KIRC, and LUAD.

Then, we used the TIMER database again to analyze the relationships between RBM8A and PD-L1 and CTLA4 in LIHC, KIRP, BLCA, KIRC, and LUAD. Our findings demonstrate that RBM8A expression has significant relationships with PD-L1 in LIHC, KIRP, BLCA, and KIRC. Additionally, RBM8A in LIHC also has significant correlations with CTLA4, while RBM8A in KIRP, BLCA, and KIRC did not have such a link. Hence, we speculate that RBM8A influences patient survival in different cancers, especially LIHC, by acting on tumor cells through immunoinfiltrating cells and immune checkpoint inhibitors. Therefore, how does RBM8A, as a small part of an exon junction complex, influence tumor immunotherapy? Whether it is direct or indirect, there are no in-depth reports exploring the relationship between immunotherapy and RBM8A.

The occurrence, proliferation, differentiation, antiapoptosis, invasion, angiogenesis, metastasis, and immune regulation of tumors are related to various signaling pathways, while the abnormal activation of signaling pathways is inseparable from the excessive activation and inhibition of a large number of cytokines and receptors. Combined with existing research, we conclude that RBM8A is an immunotherapeutic agent that acts on the signaling pathway to regulate cancer. Signal Transducer and Activator of Transcription 3 (STAT3) is a transcription factor that affects the JAK/STAT signaling pathway. When cytokines are out of regulation, the potential cancer-promoting potential of STAT3 as a proto-oncogene will continue to be expressed in cells to promote tumorigenesis [40]. Studies have confirmed that RBM8A is a STAT3 binding partner that binds to STAT3 through the C-terminal region of STAT3 in vivo and enhances IL-6-induced STAT3 activation. Furthermore, silencing RBM8A reduced IL-6-induced STAT3 tyrosine phosphorylation, nuclear accumulation, and the DNA-binding activity of STAT3, as well as IL-6/STAT3-dependent gene expression. It was demonstrated that RBM8A can interact with STAT3 and regulate transcriptional activation of STAT3 by affecting the phosphorylation of STAT3 tyrosine [41, 42]. STAT3 can be activated in tumor cells and tumor-infiltrating immune cells to further regulate the expression of oncogenes to trigger tumor progression and promote the inhibition of immune mediators [43]. STAT3 affects regulatory T (Treg) cells by inducing the expression of FOXP3 [44]. IL-10 and TGF-*β* secreted by tumor-associated regulatory T cells restrict the function of CD8+ effector T cells and the maturation of DCs, thus further inhibiting innate immunity and adaptive immunity [45, 46]. The overexpression of PD-1/PD-L1 in tumor cells is significantly related to the phosphorylation of STAT3. Targeting STAT3 can inhibit the PD-1/PD-L1 axis in an HNSCC mouse model, thus reversing the state of immunosuppression [47, 48]. In hepatoma cells, baicalein can restore the antitumor activity of T cells by reducing the activity of STAT3 and downregulating the expression of PD-L1 induced by IFN-*γ* [49]. Therefore, targeting STAT3 can not only directly inhibit tumor growth but also enhance antitumor immunity. We speculate that RBM8A plays an indirect role in tumor immunotherapy by targeting STAT3 to enhance the tumor immune response.

p53 is a transcriptional regulatory factor that mainly mediates tumor inhibition and regulates cell cycle arrest, cell apoptosis, and metabolism. Lu et al. [50] found that in different human cancer cells, the depletion of RBM8A can lead to the arrest of G2/M phase, DNA damage, and apoptosis. At the same time, it can induce the expression of another splice isoform of p53 in human cells, namely, p53*β*, and cause cell senescence. However, the increase or decrease in RBM8A will increase the level of overall p53 protein. At present, the specific molecular mechanism of the effect of RBM8A on the change in p53 is not clear. An increasing number of studies have shown that the p53 tumor suppressor signaling pathway plays an important role in the regulation of the tumor immune response [51]. For example, p53 affects the production and function of Treg cells by activating the expression of FOXP3 [52]. p53 induces the expression of DD1*α* in normal or cancer cells, resulting in the inactivation of T cells that recognize autoantigens or tumor-associated antigens [53]. p53 can also activate PD-L1 to stimulate the expression of PD-1 on the surface of T cells, resulting in immune escape [54, 55]. Therefore, we can speculate that RBM8A indirectly regulates the tumor immune response by acting on the p53 pathway.

In some studies, it was found that the p53 pathway and STAT3 pathway can interact with each other. For example, blocking the IFN-Akt pathway phosphorylates STAT3, promotes the binding of STAT3 to the p53 promoter in the nucleus, and upregulates p53. Moreover, the loss of p53 activates the JAK2/STAT3 signaling pathway and affects tumor growth [56, 57]. No related studies on RBM8A and immune response regulation have been found. In view of the above research basis and the results of mining and summarizing this study in the database, we speculate that RBM8A may act indirectly on immune checkpoints and immune-infiltrating cells through the STAT3 signaling pathway and p53 signaling pathway to affect tumor immunotherapy. At present, it is not clear whether RBM8A can directly act on immune checkpoints and immune cells in the body, which needs to be further explored in relevant experiments.

## 5. Conclusions

In summary, our study suggested that RBM8A mRNA is overexpressed in many types of cancer, and in combination with many different genes, RBM8A can influence the prognosis of cancer. RBM8A mutations are widely observed in tumors, especially missense mutations. In addition, RBM8A was strongly associated with immune-infiltrating cells and immunoassay site inhibitors, especially in LIHC, and we hypothesized that RBM8A may affect immunity through signaling pathways. Therefore, we consider that RBM8A may be a promising target in cancer immunotherapy. Although the results of RBM8A in carcinomas provide a deeper understanding of cancer immune interactions and the potential models of cancer immunotherapy, the specific mechanisms remain to be further studied.

## Data Availability

All data for this study are from an open database and are publicly available.

## Abbreviations

BLCA: Bladder urothelial carcinoma
BRCA: Breast invasive carcinoma
CESC: Cervical squamous cell carcinoma
CHOL: Cholangiocarcinoma
COAD: Colon adenocarcinoma
ESCA: Esophageal carcinoma
HNSC: Head and neck squamous cell carcinoma
LIHC: Liver hepatocellular carcinoma
LUAD: Lung adenocarcinoma
LUSC: Lung squamous cell carcinoma
UCEC: Uterine corpus endometrial carcinoma
KICH: Kidney chromophobe
KIRC: Kidney renal clear cell carcinoma
KIRP: Kidney renal papillary cell carcinoma
THCA: Thyroid carcinoma
GBM: Glioblastoma multiforme
PRAD: Prostate adenocarcinoma
PAAD: Pancreatic adenocarcinoma
PCPG: Pheochromocytoma and paraganglioma
READ: Rectum adenocarcinoma
SARC: Sarcoma
THYM: Thymoma
OS: Overall survival.

## Supplementary Materials

Supplementary Materials Supplementary Figures 1–6 and Supplementary Tables 15–20: correlation of RBM8A mutant gene expression with immune infiltration levels of macrophage, B cell, CD8+ T cell, CD4+ T cell, neutrophil, and dendritic cell in cancers (RBM8A mutant gene had no significance with the 6 infiltrated cells in the pan-cancers). Supplementary Tables 1–14: the interrelation between RBM8A and other genes in cancers.
