# Supplementary figures and images for: A Comprehensive Pan-Cancer Analysis of RBM8A Based on Data Mining
Source: J Oncol. 2021 Jul 6;2021:9983354. doi: 10.1155/2021/9983354 (PMC8277506; doi:10.1155/2021/9983354)

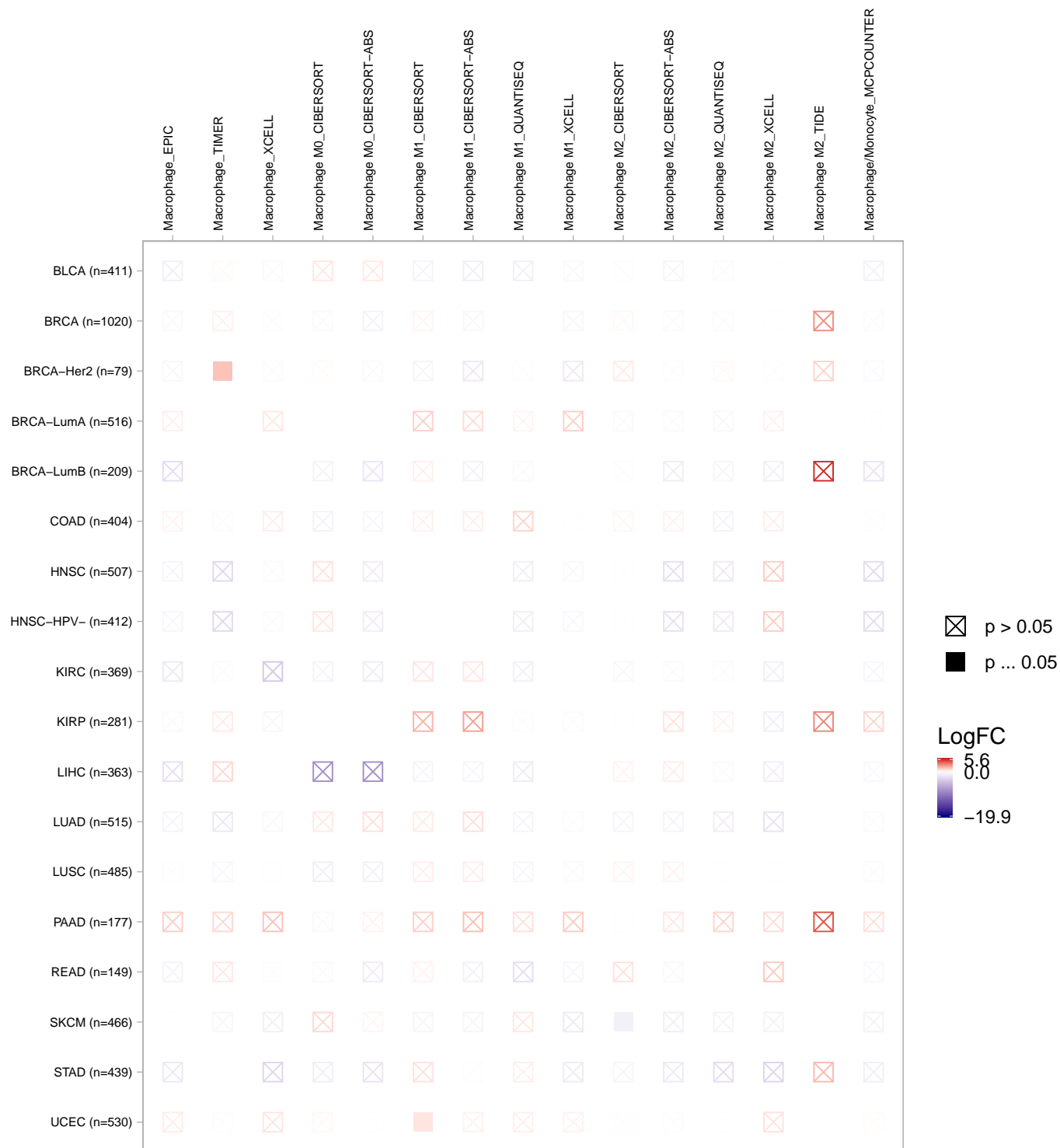

Supplement: Supplementary Materials — Supplementary Figures 1–6 and Supplementary Tables 15–20: correlation of RBM8A mutant gene expression with immune infiltration levels of macrophage, B cell, CD8+ T cell, CD4+ T cell, neutrophil, and dendritic cell in cancers (RBM8A mutant gene had no significance with the 6 infiltrated cells in the pan-cancers). Supplementary Tables 1–14: the interrelation between RBM8A and other genes in cancers. [file 9983354.f1.zip › 9983354.f1/Supplemental Figure 1 (1).pdf]

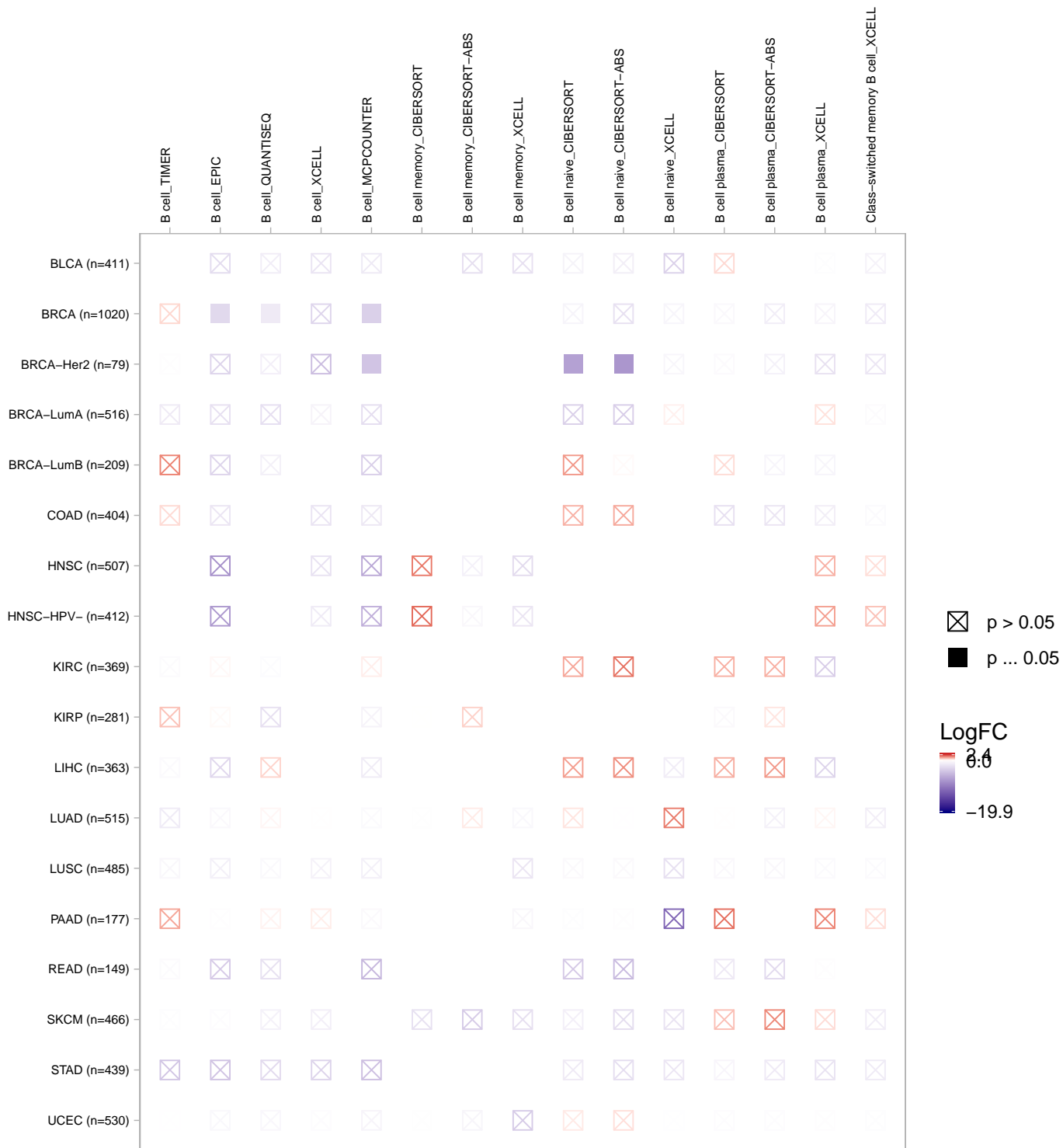

Supplement: Supplementary Materials — Supplementary Figures 1–6 and Supplementary Tables 15–20: correlation of RBM8A mutant gene expression with immune infiltration levels of macrophage, B cell, CD8+ T cell, CD4+ T cell, neutrophil, and dendritic cell in cancers (RBM8A mutant gene had no significance with the 6 infiltrated cells in the pan-cancers). Supplementary Tables 1–14: the interrelation between RBM8A and other genes in cancers. [file 9983354.f1.zip › 9983354.f1/Supplemental Figure 2 (1).pdf]

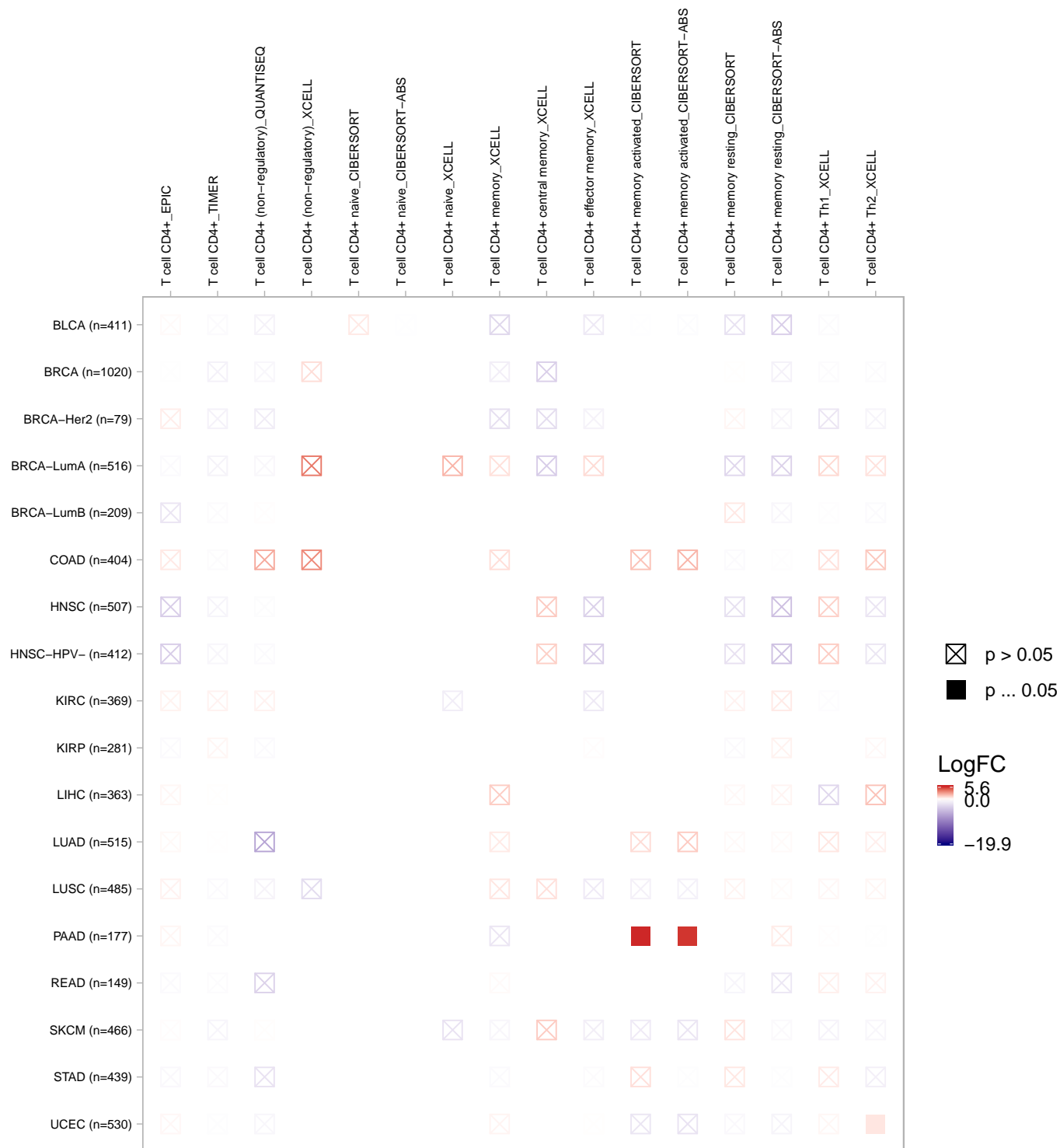

Supplement: Supplementary Materials — Supplementary Figures 1–6 and Supplementary Tables 15–20: correlation of RBM8A mutant gene expression with immune infiltration levels of macrophage, B cell, CD8+ T cell, CD4+ T cell, neutrophil, and dendritic cell in cancers (RBM8A mutant gene had no significance with the 6 infiltrated cells in the pan-cancers). Supplementary Tables 1–14: the interrelation between RBM8A and other genes in cancers. [file 9983354.f1.zip › 9983354.f1/Supplemental Figure 3 (1).pdf]

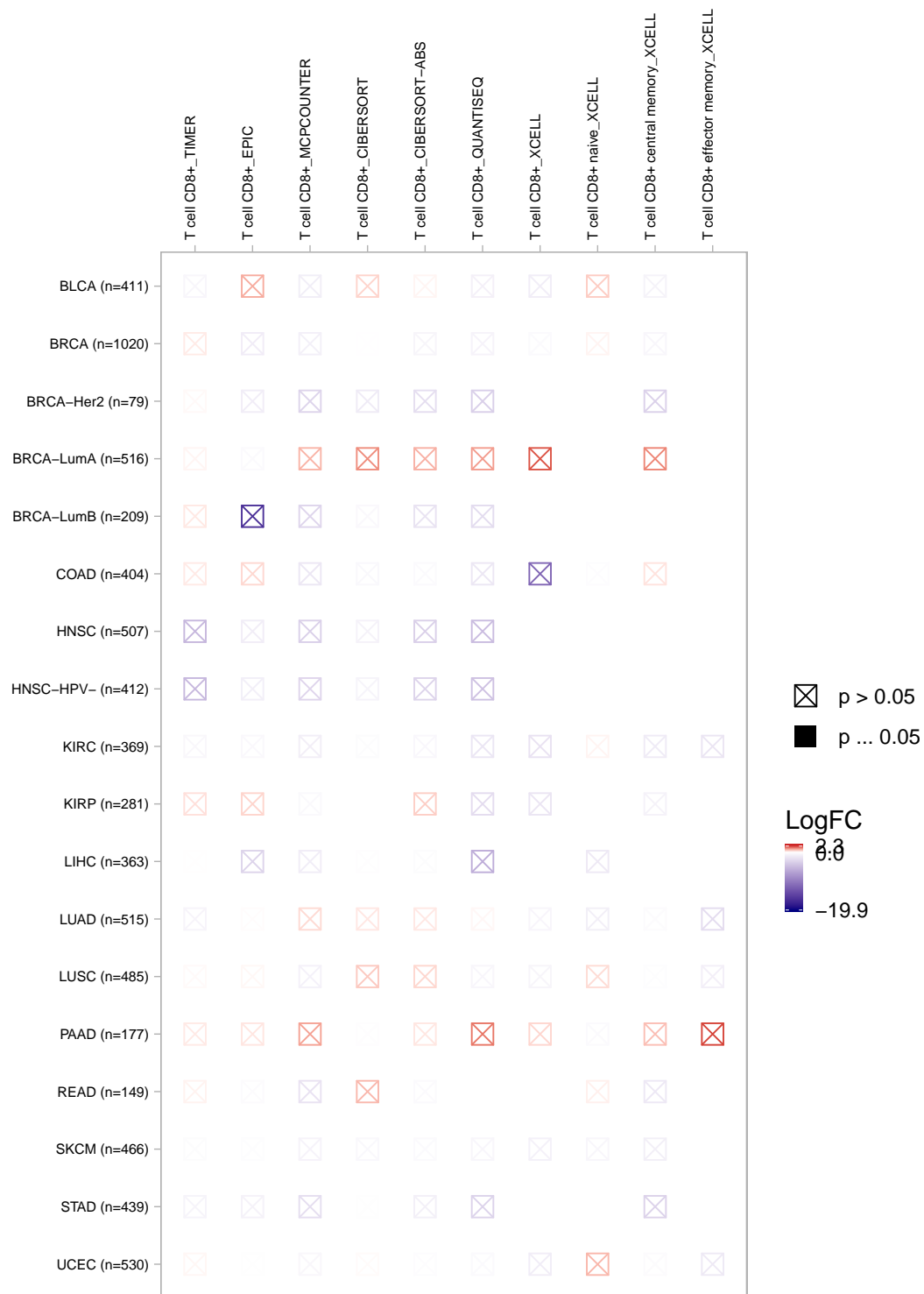

Supplement: Supplementary Materials — Supplementary Figures 1–6 and Supplementary Tables 15–20: correlation of RBM8A mutant gene expression with immune infiltration levels of macrophage, B cell, CD8+ T cell, CD4+ T cell, neutrophil, and dendritic cell in cancers (RBM8A mutant gene had no significance with the 6 infiltrated cells in the pan-cancers). Supplementary Tables 1–14: the interrelation between RBM8A and other genes in cancers. [file 9983354.f1.zip › 9983354.f1/Supplemental Figure 4 (1).pdf]

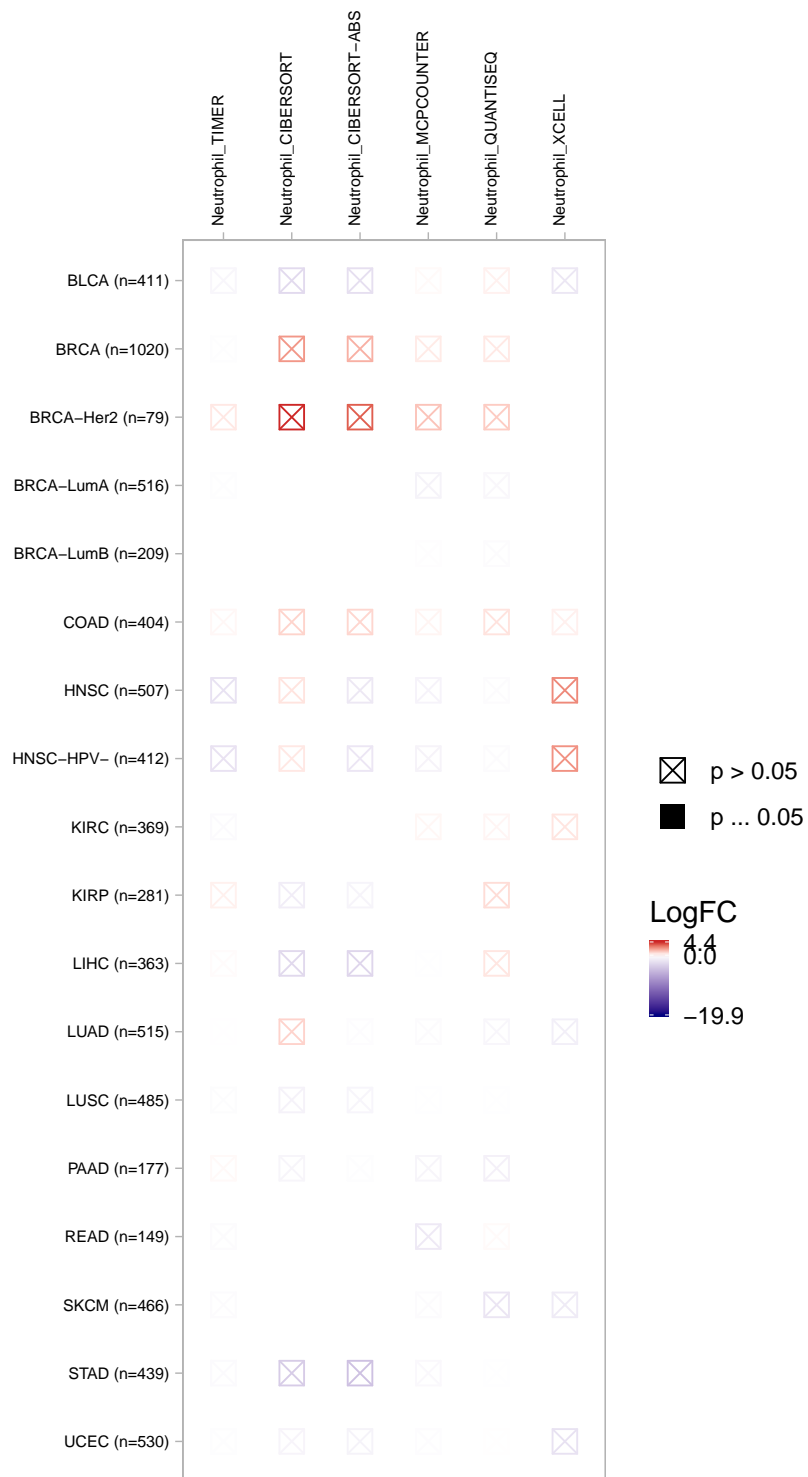

Supplement: Supplementary Materials — Supplementary Figures 1–6 and Supplementary Tables 15–20: correlation of RBM8A mutant gene expression with immune infiltration levels of macrophage, B cell, CD8+ T cell, CD4+ T cell, neutrophil, and dendritic cell in cancers (RBM8A mutant gene had no significance with the 6 infiltrated cells in the pan-cancers). Supplementary Tables 1–14: the interrelation between RBM8A and other genes in cancers. [file 9983354.f1.zip › 9983354.f1/Supplemental Figure 6 (1).pdf]
