# Supplementary material for: A Comprehensive Pan-Cancer Analysis of RBM8A Based on Data Mining
Source: J Oncol. 2021 Jul 6;2021:9983354. doi: 10.1155/2021/9983354 (PMC8277506; doi:10.1155/2021/9983354)
Supplement: Supplementary Materials — Supplementary Figures 1–6 and Supplementary Tables 15–20: correlation of RBM8A mutant gene expression with immune infiltration levels of macrophage, B cell, CD8+ T cell, CD4+ T cell, neutrophil, and dendritic cell in cancers (RBM8A mutant gene had no significance with the 6 infiltrated cells in the pan-cancers). Supplementary Tables 1–14: the interrelation between RBM8A and other genes in cancers. [file Supplemental Figure 5 (1).pdf › 9983354.f1/Supplemental Figure 5 (1).pdf]

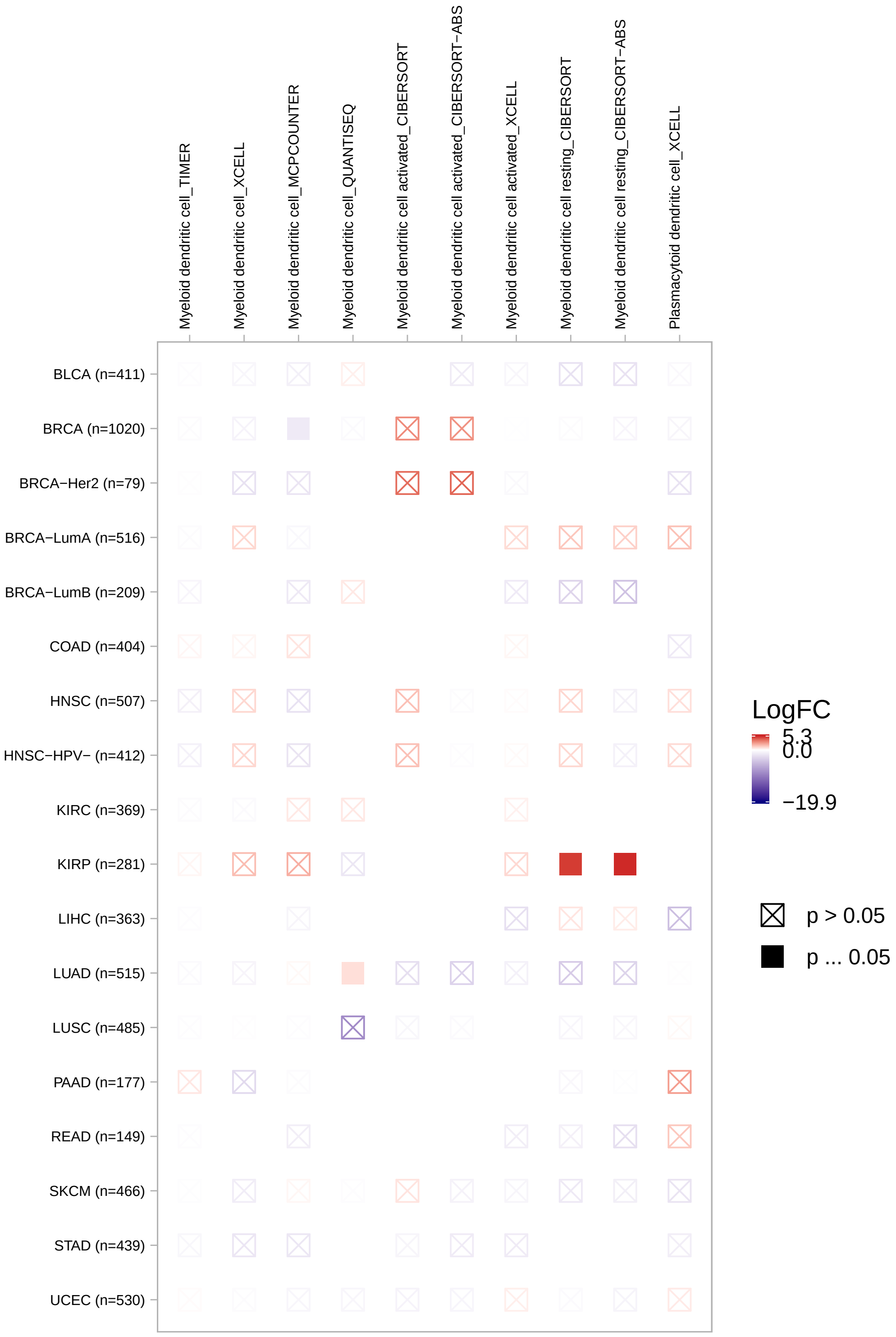

Myeloid dendritic cell_TIMER
Myeloid dendritic cell_XCELL
Myeloid dendritic cell_MCPCOUNTER
Myeloid dendritic cell_QUANTISEQ
Myeloid dendritic cell activated_CIBERSORT
Myeloid dendritic cell activated_CIBERSORT−ABS
Myeloid dendritic cell activated_XCELL
Myeloid dendritic cell resting_CIBERSORT
Myeloid dendritic cell resting_CIBERSORT−ABS
Plasmacytoid dendritic cell_XCELL
BLCA (n=411)
BRCA (n=1020)
BRCA−Her2 (n=79)
BRCA−LumA (n=516)
BRCA−LumB (n=209)
COAD (n=404)
HNSC (n=507)
HNSC−HPV− (n=412)
KIRC (n=369)
KIRP (n=281)
LIHC (n=363)
LUAD (n=515)
LUSC (n=485)
PAAD (n=177)
READ (n=149)
SKCM (n=466)
STAD (n=439)
UCEC (n=530)
LogFC
5.3
0.0
−19.9
p > 0.05
p ... 0.05
