# Supplementary material for: A Comprehensive Pan-Cancer Analysis of RBM8A Based on Data Mining
Source: J Oncol. 2021 Jul 6;2021:9983354. doi: 10.1155/2021/9983354 (PMC8277506; doi:10.1155/2021/9983354)
Supplement: Supplementary Materials — Supplementary Figures 1–6 and Supplementary Tables 15–20: correlation of RBM8A mutant gene expression with immune infiltration levels of macrophage, B cell, CD8+ T cell, CD4+ T cell, neutrophil, and dendritic cell in cancers (RBM8A mutant gene had no significance with the 6 infiltrated cells in the pan-cancers). Supplementary Tables 1–14: the interrelation between RBM8A and other genes in cancers. [file letter.pdf › 9983354.f1/letter.pdf]

Dear editor：

Thanks very much for your kind work and consideration on publication of our manuscript 9983354 entitled "A Comprehensive Analysis of RBM8A in Pan-cancer Based on Data Mining". On behalf of my co-authors, we would like to express our great appreciation to editor and reviewers.

In the part of the file I updated, because the supplementary tables cannot be uploaded in the supplementary file part, I put them in the part of uploading the picture. The figure 6 I uploaded is in editable PPT format， and the other figures I uploaded are in editable PDF format.

Thank you very much！
Yours sincerely
Chuli Li
